# Association of Spinopelvic Anatomy with the Level of Lumbar Disc Herniation

**DOI:** 10.3390/bioengineering12090993

**Published:** 2025-09-18

**Authors:** Jannis Löchel, Moritz Hanisch, Justus Bürger, Kirsten Labbus, Robert Zahn

**Affiliations:** 1 Meoclinic Berlin, MEOSPINE, Friedrichstraße 71, 10117 Berlin, Germany; 2 Center for Musculoskeletal Surgery, Charité-Universitätsmedizin Berlin, Corporate Member of Freie Universität Berlin, Humboldt-Universität zu Berlin, and Berlin Institute of Health, 13353 Berlin, Germany; justus.buerger@icloud.com (J.B.); kirsten.labbus@charite.de (K.L.); 3 Clinic for Orthopaedics, Ulm University Hospital, Campus Nord Oberer Eselsberg, Oberer Eselsberg 45, 89081 Ulm, Germany

**Keywords:** spinopelvic alignment, spinopelvic anatomy, lumbar disc herniation, level

## Abstract

Aim: The aim of this study was to investigate the association between the level of lumbar disc herniation (LDH) and individual spinopelvic anatomy. Material and methods: Spinopelvic parameters were retrospectively evaluated in 57 patients with symptomatic LDH at L4/5 and L5/S1 undergoing minimal invasive sequestrectomy at our institution. LDH was diagnosed in 23 patients at L5/S1 and in 34 patients at L4/5. Patients with further segment degeneration at the index level were excluded from the study. Results: Spinopelvic parameters between the two groups were significantly different. Patients with LDH at L5/S1 had statistically significant lower Pelvic Incidence (PI), Pelvic Tilt (PT), Relative Lumbar Lordosis (RLL) and PI-LL than patients with LDH at L4/5. C7 Sagittal Vertical Axis (C7SVA) was statistically significant lower in patients with LDH at L5/S1. Both groups had no sagittal imbalance. Patients with LDH at L5/S1 were significantly younger than patients with LDH at L4/5. There was a significant positive correlation between age and PT. We observed no significant differences for preoperative values of Lumar Lordosis (LL) and Sacral Slope between the two groups. Conclusions: This is the first study to reveal individual spinopelvic anatomy and, in particular, PI to be associated with the distinct level of LDH. These findings substantiate the biomechanical influence of the sagittal profile on the pathogenesis of LDH. Individual spinopelvic compensatory mechanisms were available independently of the patient’s age. Minimal invasive sequestrectomy is a reliable treatment for symptomatic LDH without further segment degeneration.

## 1. Introduction

Lumbar spinal degeneration is a common condition in the aging population. Degenerative disc disease (DDD) and lumbar disc herniation (LDH) represent early manifestations of segmental degeneration. The progression of spinal degeneration can lead to more complex pathologies such as osteochondrosis (OC), spondylarthrosis, spinal stenosis, spondylolisthesis, and adult spinal deformities [1].

Despite significant advancements in understanding the genesis of several spinal pathologies, the specific impact of individual spinopelvic anatomy and resulting biomechanical factors on the development at the most affected levels of LDH has not been investigated in detail until now.

The influence of spinopelvic anatomy and biomechanics is increasingly recognized as a determinant in the development and progression of degenerative spinal conditions [2,3]. Among the parameters of the spinal sagittal profile, Pelvic Incidence (PI) stands out as the invariable anatomical parameter, influencing the overall spinal shape and load distribution [4,5]. Prior research has demonstrated the influence of PI, along with other parameters such as Pelvic Tilt (PT), Sacral Slope, Lumbar Lordosis (LL), and C7 Sagittal Vertical Axis (C7SVA), on maintaining sagittal balance and adapting to spinal pathologies [4,5,6,7,8]. A high PI with high LL leads to a higher load on posterior parts of the spine and was observed to be associated with an increased risk for the development of spondylarthrosis and degenerative spondylolisthesis [2,3,7,9,10]. In comparison, the occurrence of DDD or OC is conditioned by higher load on anterior parts of the spine at low PI and minor LL [5,11,12]. In a previous study, it could be shown that PI plays an important role concerning the level of occurrence and severity of isthmic spondylolisthesis (iSPL) and that spinopelvic anatomy determines the pathogenesis of iSPL [13].

While PI is a static anatomical parameter, the spinopelvic alignment is dynamic and capable of compensatory mechanisms to preserve sagittal balance in degenerative spinal pathologies, including LDH [14]. A recent longitudinal study showed that the magnitude of PI significantly influences compensatory mechanisms associated with age-related deterioration of spinopelvic alignment [15].

The pathogenesis of LDH is multifactorial and the most affected segments are L4/5 and L5/S1 in 90% [16,17,18,19]. An association between a low PI and the occurrence of LDH in younger individuals and a trend towards the affection of upper lumbar segments in older patients have been observed [5,12,20,21,22]. Knowledge of the influence of individual spinopelvic interactions on the manifestation of LDH would give a more detailed understanding of the complex interactions between individual anatomy and resulting biomechanics of the spine in a common spinal pathology.

The aim of this study was therefore to investigate the association between spinopelvic anatomy with a comprehensive set of spinopelvic parameters including PI, PT, and Sacral Slope and the distinct level of LDH.

## 2. Materials and Methods

### 2.1. Study Design

The study was designed as a single-center, retrospective clinical study. Patients with symptomatic LDH at L4/5 and L5/S1 who underwent minimal invasive sequestrectomy at our institution (university hospital) between 2016 and 2023 were included in this study. Surgical intervention was specifically indicated for patients experiencing radicular symptoms due to LDH that had proven unresponsive to a period of comprehensive conservative treatment. To maintain a clear focus on isolated LDH and its relationship with spinopelvic anatomy, patients with any evidence of further degenerative changes at the index level were excluded from the study. Additional exclusion criteria encompassed a history of previous spinal surgery, multilevel LDH, spinal tumor, spinal trauma, or presentation as an emergency surgery, all of which could confound the analysis of inherent spinopelvic influences on LDH pathogenesis.

The study was approved by the local ethics committee prior to the initiation (EA 1/342/21).

### 2.2. Radiographic Analysis

Diagnosis of LDH was routinely confirmed by preoperative magnetic resonance imaging (MRI). To assess global spinal alignment and individual spinopelvic morphology, all patients underwent standardized preoperative standing lateral full spine radiographs. Patients were positioned with their knees extended and arms supported during radiography. This protocol was specifically designed to ensure that radiographs were reproducible across examinations and to allow for pre- and postoperative comparative measurements. Spinopelvic parameters as defined by PT, PI, Sacral Slope, LL, C7SVA, PI-LL were manually measured preoperatively using the SurgiMap Spine software version 2.3.2.1 (Nemaris Inc.; New York, NY, USA). To maintain measurement consistency and reliability, all parameters were assessed by a single experienced spine surgeon. The methodologies for measuring each parameter were precisely defined: PI was assessed with the angle formed by a perpendicular line from the midpoint of the sacral endplate and a line connecting this midpoint to the center of the femoral head. PI represents a fixed anatomical pelvic shape. PT was defined as the angle between the plumb line and the line connecting the center of the femoral heads to the midpoint of the sacral endplate. The Sacral Slope was measured as the angle between superior endplate of the S1 vertebra and a horizontal reference line. LL was measured using the Cobb angle method. LL was measured as the angle between the superior endplate of L1 and the superior endplate of S1. C7SVA was established as the horizontal distance between a plumb line dropped from the center of the C7 vertebral body and the posterosuperior corner of the S1 vertebral body, serving as an indicator of global sagittal balance. Additionally, PI-LL mismatch was calculated as the absolute difference between PI and LL (PI-LL). Relative Lumbar Lordosis (RLL) was calculated by subtracting measured LL from ideal LL, with ideal LL being dependent on the patient’s pelvic incidence (PI). RLL was calculated using this formula: RLL = L1 − S1 Lordosis minus (0.62 × PI + 29) [23,24]. All measurements were performed by an experienced spine surgeon using the SurgiMap Spine software (Nemaris Inc.; New York, NY, USA). Previously high intra- and interobserver reliability was shown for all sagittal parameters [3,6,7,25].

### 2.3. Statistical Analysis

Descriptive statistics were calculated for all demographic and radiographic parameters. Continuous variables were presented as mean standard deviation, while categorical variables were expressed as frequencies and percentages. We used the Kolmogorov–Smirnov test to assess normal distribution of data. To facilitate comparisons between the two groups (L4/5 LDH vs. L5/S1 LDH) independent samples t-tests were employed for normally distributed continuous variables. For non-parametric data we used the Mann–Whitney U test. For evaluation of associations between continuous variables Spearman’s rank correlation coefficient was utilized. A *p*-value of < 0.05 was considered statistically significant. All statistical analyses were performed using SPSS Statistics software, Version 27 (IBM Corp., Armonk, NY, USA).

## 3. Results

A total of 57 patients was included in the study, of whom 32 were women and 25 were men. The overall mean age of the study population was 48.0 ± 12.8 years. Group allocation was performed according to the level of the affected spinal segment.

The L5/S1 group consisted of 23 patients, accounting for 40.4% of the total patient collective. The mean age of individuals in this group was 43.4 ± 12.4 years. Sex distribution within the L5/S1 group was composed of 13 women (56.5%) and 10 men (43.5%). The L4/L5 group included 34 patients (59.6%). Patients in this group had a mean age of 50.0 ± 11.7 years including 19 (55.9%) women and 15 (44%) men.

The mean age for men in the L4/L5 group was 53.4 years, compared to 43.9 years in the L5/S1 group. For women, the mean age was 47.3 years in the L4/L5 group and 42.9 years in the L5/S1 group. The distribution of sex did not differ significantly between the L4/L5 and L5/S1 groups (χ^2^ = 0.002, *p* = 0.962). There were no significant differences between age distribution in both groups calculated with the Mann–Whitney U Test (L5/S1—*p* = 0.56, U = 74.5; L4/L5—*p* = 0.07, U = 193.5) The detailed analysis of preoperative radiographic parameters according to group is listed in Table 1.

Diagram 1. Box plot diagram for L4/5 and L5/S1 group comparison for selected spinopelvic parameters given in degrees. 
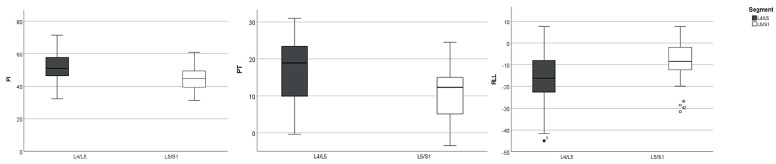


**Figure 1:**
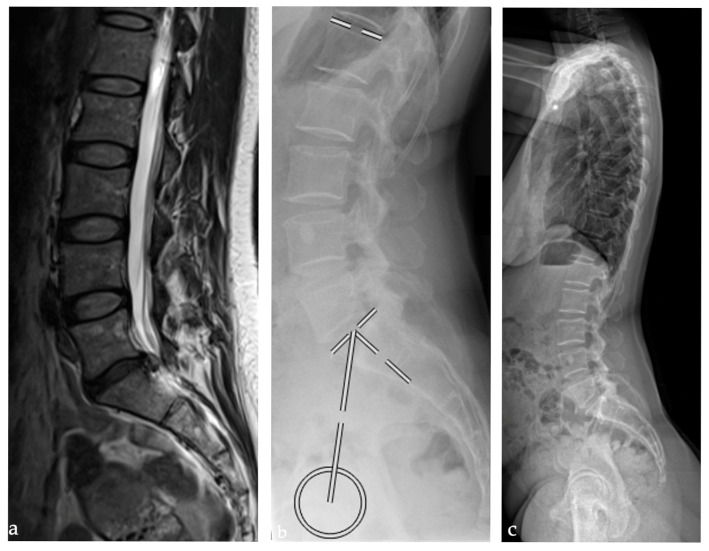
Patient example illustrating lumbar disc herniation at L5/S1 and mean spinopelvic parameters: pelvic incidence: 44.7°, lumbar lordosis: 48.2°, and relative lumbar lordosis: −8.6° displayed in an (**a**) MRI (**b**) lateral lumbar spine radiograph (**c**) standing lateral full spine radiograph.

**Figure 2:**
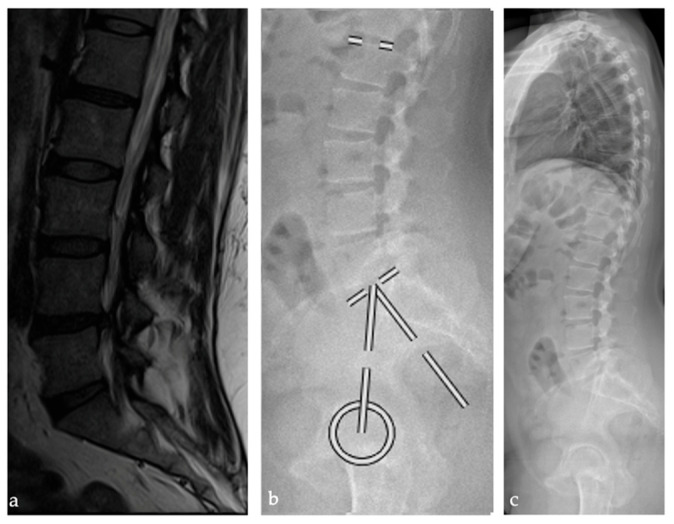
Patient example illustrating lumbar disc herniation at L4/L5 and mean spinopelvic parameters: pelvic incidence 52.1°, lumbar lordosis 45.8° and a relative lumbar lordosis of −15.5° displayed in an (**a**) MRI (**b**) lateral lumbar spine radiograph (**c**) standing lateral full spine radiograph.

The mean PI of patients with LDH at L5/S1 was 44.7 ± 8.0° and was statistically significantly lower compared to the PI of 52.1 ± 9.2° for patients with LDH at L4/5 (*p* = 0.004). Similarly, the mean PI of patients with LDH at L5/S1 was significantly lower with 11 ± 8° compared to patients of the L4/5 group with 17.8 ± 8.3° (*p* = 0.006). The RLL showed significantly lower values for the L4/5 group (−15.5 ± 12.4°) compared to patients of the L5/S1 group (−8.6 ± 9.6°; *p* = 0.026). The PI-LL mismatch was significantly lower in the L5/S1 group, compared to patients with LDH at L4/5 (Spearman Rho Correlation Index= −0.380, *p* = 0.004). C7SVA was significantly lower for patients with LDH at L5/S1 (22.7 ± 41.7 mm) compared to patients with LDH at L4/5 (49 ± 41.5 mm; *p* = 0.019), whereas both groups had no sagittal imbalance with C7SVA greater than 100 mm. Patient age correlated positively with PT (Spearman Rho Correlation Index= −0.298, with *p* = 0.026). We observed no significant differences for Sacral Slope and LL between the two groups. A Mann–Whitney U Test was performed for subgroup analysis. The subgroup analysis by sex showed no significant differences for all spinopelvic parameters for the L4/5 group. In the L5/S1 group female patients showed significantly higher Sacral Slope compared to men (*p* = 0.022, effect size 0.51). There were no further significant sex-associated differences in this group. Figure 1 and Figure 2 illustrate the routinely performed preoperative diagnostics and workup.

## 4. Discussion

In this study, we investigated the association between the distinct level of LDH and spinopelvic anatomy. For the first time, it could be shown that the distinct level of LDH is associated with the extent of PI.

The influence of spinopelvic anatomy and biomechanics on the genesis of advanced patterns of spinal degeneration has been shown previously [2,26,27]. To our knowledge, there is no previously published study investigating the association between the distinct level of LDH as a pathology of initial segment degeneration and sagittal profile.

The etiology of LDH is multifactorial. Genetic predisposition, advancing age, sex, obesity, and specific lifestyle elements have been identified as risk factors for the development of LDH [1,4,16,18,19,27]. However, our findings emphasize the clinically relevant impact of spinopelvic anatomy and biomechanics on the pathogenesis of LDH.

In previous studies, a low PI and the sagittal profile types I and II of the Roussouly classification were identified as a risk factor for the development of LDH in younger patients [20,22].

In our study, LDH was likewise observed in patients with a low PI and spinopelvic anatomy according to Roussouly types I and II [1,4,5]. Types I and II of the Roussouly classification are characterized by Sacral Slopes values < 35 degrees associated with low to moderate PI. These types showed reduced load-bearing capacity and higher intradiscal pressures in movement in a finite element model [28]. The results of our clinical study support these findings. Just as LDH, symptomatic iSPL most commonly occurs in L5/S1 and L4/5 [29]. A previous study revealed the biomechanical impact of spinopelvic anatomy and, in particular, PI on the pathogenesis of iSPL. Patients with iSPL at L5/S1 had significantly higher PI values than patients with iSPL at L4/5. A higher PI could lead to increased shear stress in the predisposed segment and condition the observed higher grade of slippage of iSPL in L5/S1 [13].

These findings all correspond to the model of sagittal profile with the relevant impact of spinopelvic biomechanics in the development of spinal pathologies.

A low PI associated with low LL may predispose individuals to LDH due to increased anterior loading and elevated pressure on the intervertebral discs [5,30]. A high PI with increased LL may lead to iSPL as a result of elevated shear forces, which can promote facet joint degeneration and vertebral slippage [2,9,10,31,32]. These results and the results of previous studies indicate that specific mono- or multisegmental spinal pathologies are influenced and conditioned by distinct spinopelvic biomechanical forces.

A high PI and corresponding high LL allow for greater spinopelvic compensation mechanisms [3,33]. Despite significant differences in PI between the two groups in this study, there was no statistically significant difference in the preoperative mean values of LL between the two groups. This finding suggests the existence of a different capacity for compensation mechanisms. Patients with higher PI and LDH at L4/5 presented increased compensatory adaptation. These findings highlight the importance of RLL when assessing LL in the context of sagittal balance in patients with degenerative lumbar pathologies. Compensatory mechanisms have been observed in other degenerative spinal pathologies before and are suggested to exist in patients with LDH. The reduction in lordosis relative to the individual spinopelvic anatomy proves the existence of compensatory mechanisms in patients with LDH [34]. One of the key mechanisms of compensation is an increased PT. This is concordant with other studies investigating compensatory mechanisms in patients with chronic lower back pain [5]. Interestingly, patients with LDH at L4/5 were significantly older than patients with LDH at L5/S1. This supports previously published results, showing a trend towards more cranial segments for LDH occurrence with increasing age [21].

The findings of our study demonstrate the existence of compensatory mechanisms in older patients with LDH. The age difference between the two groups emphasizes the importance of biomechanics of the spine on the pathogenesis and compensatory adaptations after LDH.

Adaptation to degenerative spinal disorders and compensatory mechanisms are in focus of actual musculoskeletal research. A recently published study demonstrated that individuals with high PI compensate by decreasing thoracic kyphosis, while those with low PI show an increase in LL. This aligns with our observation that patients with LDH at L5/S1, which are associated with lower PI, exhibited reduced compensatory reduction in LL compared to those with L4/5 LDH with higher PI. Compensatory mechanisms are shaped by individual spinopelvic parameters. These findings proof that even in early degenerative conditions like LDH, dynamic compensatory mechanisms exist, allowing patients to maintain global sagittal balance [15]. All patients of the study had a SVA < 10 mm and therefore no sagittal imbalance, which can even be observed in LDH patients [35].

The comparison between female and male patients showed no statistically significant differences for the analyzed spinopelvic parameters except for a higher Sacral Slope for women in the L5/S1 group. Compensatory changes in sagittal profile in LDH influence the variable parameters of sagittal profile masking underlying sex differences. These compensatory mechanisms do not include PI, as a fixed anatomical parameter.

Limitations of this study can be seen in the retrospective design and the relatively small study population. The small study population resulted from the strict exclusion of patients with further degeneration of the affected segment and the exclusive consideration of surgically treated patients. The homogeneous cohort selection allowed focused analysis without the confounder of segment degeneration. Future longitudinal studies are needed to evaluate the long-term biomechanical influence of spinopelvic parameters on the genesis of specific spinal pathologies and their clinical implications.

## 5. Conclusions

This study provides novel insights into the pathogenesis of LDH, demonstrating for the first time a significant association between the distinct level of LDH and individual spinopelvic anatomy, particularly PI. Our findings indicate that specific inherent sagittal profiles may predispose individuals to LDH at the L4/5 versus the L5/S1 segment. PI differs between individuals with LDH at L4/5 compared to L5/S1. Compensatory mechanisms in patients with LDH exist independently of age. These findings underscore the relevant biomechanical influence of the sagittal profile on the development of LDH and highlight the clinical relevance of understanding individual spinopelvic anatomy. While sequestrectomy remains a reliable and commonly practiced treatment for symptomatic LDH without further segment degeneration, our knowledge of the underlying biomechanical predispositions could pave the way for establishing more targeted preventive measures for LDH in the future.

## Figures and Tables

**Table 1:** Preoperative spinopelvic parameters. PI: pelvic incidence; PT: pelvic tilt; SS: sacral slope; LL: lumbar lordosis; RLL: relative lumbar lordosis; SVA: sagittal vertical axis. Standard deviation (±); *p*: statistical significance of values.

Segment		PI [°]	PT [°]	SS [°]	LL [°]	RLL [°]	SVA [mm]
L4/5 (*n* = 34)	Mean	52.1 (±9.2)	17.8 (±8.3)	34 (±9.7)	45.8 (±14.4)	−15.5 (±12.4)	49 (±41.5)
L5/S1 (*n* = 23)	Mean	44.7 (±8)	11 (±7.2)	33.6 (±7.3)	48.2 (±9.3)	−8.6 (±9.6)	22.7 (±41.7)
	*p*	0.004	0.006	0.67	0.33	0.026	0.019
Mean overall (*n* = 57)	Mean	50 (±9.8)	15.9 (±9.6)	34.3 (±8.8)	46.8 (±13)	−13.3 (±12.6)	39.4 (±43)

## Data Availability

The data are not publicly available due to privacy.

## Abbreviations

The following abbreviations are used in this manuscript:

PI	Pelvic Incidence
PT	Pelvic Tilt
LL	Lumbar Lordosis
SS	Sacral Slope
RLL	Relative Lumbar Lordosis
SVA	Sagittal Vertical Axis
iPSL	Isthmic spondylolisthesis
C7SVA	C7 Sagittal Vertical Axis
LDH	Lumbar disc herniation
